# Smoking Promotes AT2 Cell Senescence and Exacerbates Pulmonary Fibrosis by Downregulating POT1 via Integratively Inducing CpG Methylation and MECP2‐Mediated FOXP2 Transcriptional Binding Inhibition

**DOI:** 10.1111/acel.70174

**Published:** 2025-07-20

**Authors:** Mengkun Shi, Wei Wang, Posum Wan, Jialun Shi, Huixia Cui, Zhonghan Sun, Xiaofeng Chen, Jingyu Chen, Jiucun Wang, Xiangguang Shi

**Affiliations:** ^1^ Department of Thoracic Surgery, Huashan Hospital & Cancer Metastasis Institute Fudan University Shanghai China; ^2^ School of Life Sciences Fudan University Shanghai China; ^3^ Wuxi Laboratory of Organ Transplantation Wuxi People's Hospital Affiliated to Nanjing Medical University Wuxi China; ^4^ Department of Cardiothoracic Surgery, Heping Hospital Changzhi Medical College Changzhi Shanxi China; ^5^ Department of Medical Institution Conducting Clinical Trials for Human Used Drug, Heping Hospital Changzhi Medical College Changzhi Shanxi China; ^6^ State Key Laboratory of Genetic Engineering, Human Phenome Institute, School of Life Sciences Fudan University Shanghai China; ^7^ Wuxi Lung Transplant Center Wuxi People's Hospital Affiliated to Nanjing Medical University Wuxi China; ^8^ Center for Lung Transplantation, Second Affiliated Hospital Zhejiang University School of Medicine Hangzhou China; ^9^ Department of Dermatology, Huashan Hospital Fudan University Shanghai China

**Keywords:** cell senescence, CpG methylation, POT1, pulmonary fibrosis, smoking

## Abstract

Smoking is one of the most recognized risk factors for pulmonary fibrosis (PF). However, the underlying mechanism is not well understood. This study reveals smoking increases the risk of developing idiopathic PF (IPF) and that smoked IPF patients exhibit higher levels of senescence markers than non‐smoker IPF patients. Moreover, smoking enhances bleomycin (Bleo)‐induced PF, along with obvious senescence of type II alveolar (AT2) cells. RNA‐seq assay identifies cigarette downregulates protection of telomeres 1 (POT1), which is then validated to decrease in smoked PF patients and mice via upregulating the methyltransferase MECP2. Mechanistically, MECP2 binds to the DNA methyltransferases (DNMTs)‐induced methylated CpG island in the POT1 promoter, and smoking inhibits the transcriptional activity of the CpG island. The transcription factor FOXP2 could bind to this CpG island to promote POT1 transcription. However, this process is inhibited by forming a MECP2−FOXP2 complex, which blunts the FOXP2−POT1 DNA binding. siRNA‐mediated *POT1* knockdown promoted AT2 cell senescence in a p‐ATM and p‐ATR‐dependent manner and secreted inflammatory and profibrotic factors, further promoting fibrotic response in fibroblasts. In vivo, delivery of the adeno‐associated virus 9‐POT1 (AAV9‐POT1) vector inhibits cigarette‐induced cell senescence and effectively alleviates PF in mice. These findings demonstrate that POT1 is an essential protector in PF by protecting against AT2 cell senescence.

## Graphical abstract

Smoking is one of the major causes of pulmonary fibrosis (PF), and treatment options for PF are limited. We show that POT1 is decreased in PF patients and mice, especially in smoke exposure, and investigate its contributions to PF progression from protecting AT2 cell senescence to fibrosis. AAV9‐mediated restoration of POT1 prevents PF.

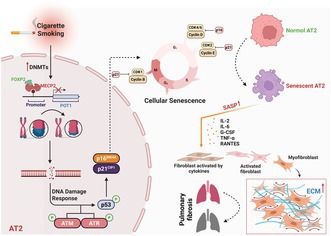

## 1 Introduction

Pulmonary fibrosis (PF) is a highly fatal disease characterized by irreversible structural damage, fibrous hyperplasia, and respiratory failure (Moss et al. 2022). Pulmonary fibrosis has many causes and can occur spontaneously or secondarily to various lung and other tissue diseases. PF encompasses several etiological subtypes, including IPF, connective tissue disease represented by scleroderma‐related PF, silicosis, smoking‐induced PF, virus‐induced PF, familial IPF, and cystic fibrosis caused by mutation of the CFTR gene (Shi et al. 2024).

The cumulative global death toll from smoking could reach one billion this century, most of them in low‐ and middle‐income countries, including China. Smoking increases the risk of 22 causes of death and 56 diseases, including infectious, cancer, metabolic, neurological, eye, cardiovascular, respiratory, and esophageal diseases (Chan et al. 2022). There is ample evidence that smoking can cause chronic obstructive pulmonary disease, respiratory infections, tuberculosis, and a variety of interstitial lung diseases, and the more you smoke and the more years you smoke, the greater the risk of disease. In a recent study, results from a nationwide population‐based cohort study that identified a total of 25,113 individuals (0.11%) with incident IPF among 23,242,836 participants showed that smoking significantly increased the risk of developing IPF. Current smokers had a higher risk of IPF than former smokers (Bae et al. 2022). A prospective cohort study from the UK Biobank showed that both active and maternal tobacco smoking have an independent and synergistic adverse effect on the risk of IPF. Moreover, smoking intensity also shows a dose–response relationship with IPF, strengthening the hypothesis of a potential causal relationship (Bellou et al. 2021). However, although smoking can be a cause or aggravator of pulmonary fibrosis, the mechanism is not fully understood.

Cellular senescence plays a vital role in the pathogenesis of pulmonary fibrosis. Studies have shown that senescent cells accumulate in large numbers in fibrotic lungs and that pulmonary fibrosis can be attenuated using senolytics (Lehmann et al. 2017; Nambiar et al. 2023; Schafer et al. 2017; Zhou et al. 2019). Alveolar epithelial cells (AECs) are essential for maintaining lung function stability and are also the central target cells of lung aging (Chin et al. 2023). AEC senescence is a common pathological mechanism in many chronic lung diseases (Barnes 2016; Liu et al. 2024; Wang et al. 2020). Single‐cell transcriptome analysis also revealed many senescent AECs in the lungs of patients with idiopathic pulmonary fibrosis (Reyfman et al. 2019; Yao et al. 2021). Elimination of senescent cells can inhibit pulmonary fibrosis (Parimon et al. 2023; Zhang et al. 2023). Therefore, targeting senescent AECs is critical for treating age‐related chronic lung disease.

POT1 is part of the shelterin complex and is essential for regulating and maintaining telomere length, and telomere shortening is an important biomarker of cell senescence (Lopez‐Otin et al. 2023). Moreover, telomere shortening has been reported to be involved in developing pulmonary fibrosis. For instance, in the UK Biobank, a one SD shorter telomere length was associated with an increased likelihood of IPF (odds ratio [OR] 4.19, 95% CI 2.33–7.55; *p* = 0.0031). A similar association was found in the IPF replication cohort (12.3, 5.05–30.1; *p* = 0.0015; Duckworth et al. 2021). Telomere shortening causes alveolar cell failure, including alveolar stem cell failure (Alder et al. 2015). In addition, there is evidence that smoking may lead to shorter telomeres (Astuti, Wardhana, Watkins, Wulaningsih, and Network 2017). Smoking also involves lung epithelial cell senescence through the Sp1/SIRT1/HIF‐1α pathway (Wu et al. 2022). These findings suggest that POT1 is essential in inhibiting telomere shortening and may protect against cellular senescence and pulmonary fibrosis induced by adverse factors, including smoking.

In this study, we found that smoking increased the IPF ratio, promoted AT2 cell senescence, and downregulated POT1. In vivo, prolonged smoke exposure significantly induced PF in mice and exacerbated Bleo‐induced PF. Mechanistically, smoking inhibits POT1 expression by upregulating DNMTs‐induced DNA methylation of the *POT1* CpG island. In addition, the protein interaction between MECP2 and FOXP2 further suppressed the transcriptional activation of FOXP2 on POT1. POT1 downregulation induces senescence of AT2 cells, which not only directly causes structural damage to the lung but also secretes large amounts of SASP to activate fibroblasts, ultimately leading to the formation of pulmonary fibrosis. Restoring the expression of POT1 can reverse the above phenotype.

## 2 Materials and Methods

### 2.1 Human Specimens

Jingyu Chen and the UGMLC Giessen Biobank generously provided lung samples from patients with IPF and control subjects. Laboratory testing confirmed IPF. Detailed clinical information is shown in Table S1. All subjects provided informed consent before participation and were briefed regarding the study. This research received approval from the School of Life Sciences at Fudan University (Approval no. KY2023‐015).

### 2.2 Construction of Cigarette‐ and Bleo‐Induced PF Mouse Model

The Fudan University Animal Care and Use Committee approved every animal experiment used in this work. Male C57BL/6J mice aged six (4–6) weeks, weighing 19 ± 1 g, were acquired from Shanghai SLAC Laboratory Animal Co. Ltd. Male mice aged 6 weeks were intraperitoneally injected with 2.0 mg/kg Bleo (diluted in sterile saline) or the same quantities of saline according to the prior protocol. Mice in the cigarette smoking groups were housed in a 0.1 m^3^ plexiglass smoke chamber to simulate cigarette smoke exposure in animal models. The commercial cigarettes (16 mg tar, 1.0 mg nicotine per cigarette) were ignited, and an air pump was used to introduce smoke into the chamber. For the first 7 days, mice were fed in adaptive feeding. Then, from Days 8 to 68, mice were exposed to smoke for approximately 1 h per day, 5 days a week, with a total of 12 cigarettes per day. In a similar chamber, control animals were exposed to ambient air. On Day 47, mice received an intratracheal instillation of a single dose of 2 mg/kg bleomycin or saline. On Day 68, all mice were put to death, and lung tissues were used in lab tests and histopathology. The degree of lung fibrosis was assessed by HE and Masson, as well as laboratory testing.

### 2.3 Histological Analysis

The degree of fibrosis and inflammation was evaluated using Masson's trichrome staining and H&E. Slides were rated using the Ashcroft score system on a scale of 0 to 8. A Nikon Eclipse80i microscope (Nikon) was used to image the sections at magnification.

### 2.4 Gene Expression Analysis

Using an M‐MLV First Strand Kit (Life Technologies), total RNAs extracted from lung tissues using TRIzol reagent were exposed to the reverse transcriptase process to create cDNA. A Roche LC480 Real‐Time PCR System (Applied Biosystems) was used to conduct quantitative real‐time PCR (qRT‐PCR) utilizing the SYBR Green technique. To GAPDH or β‐actin, relative mRNA levels were adjusted. Primer sequences are listed in Table S2.

### 2.5 Western Blot Analysis

Proteins were isolated from the whole lung lysate, AT2, and fibroblast cells. The proteins were separated using 10% SDS‐PAGE and then put onto nitrocellulose membranes. After blocking the non‐specific proteins with 5% BSA, incubate the membranes for 2 h at room temperature with HRP‐conjugated secondary antibodies and an overnight stay at 4°C with primary antibodies. An ECL kit (Pierce, Rockford, IL, USA) was utilized to find protein bands. To measure protein levels, β‐actin or GAPDH was used as a normalization. The antibodies employed in this investigation are listed in Table S3.

### 2.6 Collagen Measurements

The Sircol collagen assay (Biocolor, Belfast, UK) was employed to detect the amount of soluble collagen in the lungs. This determination was carried out following the manufacturer's protocol. The collagen measurements were subsequently normalized to the total protein content, which was determined using a BCA protein assay kit (Beyotime, Nanjing, China).

### 2.7 General Cell Culture

Primary fibroblast cells were obtained from patients with PF (PF). Following FAC sorting, these primary fibroblasts were cultured in Dulbecco's Modified Eagle Medium (DMEM) supplemented with 10% fetal bovine serum (FBS). The primary human lung endothelial and AT2 cells were seeded in Fibronectin‐coated plates and cultured in Dulbecco's Modified Eagle Medium (DMEM) supplemented with 10% FBS. All cells were maintained at 37°C in a humidified incubator with 5% CO_2_. *si‐POT1* was initially incubated with pHLAT2 cells for 48 h to collect a conditioned medium. Subsequently, the conditioned medium was collected and stored at −80°C for later stimulation of primary fibroblast cells.

### 2.8 Immunohistochemical Staining

To prepare the lung tissues for the immunohistochemistry assay, they were deparaffinized and then incubated with 5% bovine serum albumin for 60 min. P21‐positive and COL1A1‐positive cells were detected by adding the primary antibody (P21, Abclonal, A22460; COL1A1, Abclonal, A1352; POT1, MECP2) and incubating for 2 h at room temperature. This was followed by incubation with 3% hydrogen peroxide for 10 min. As secondary antibodies, we employed goat anti‐rabbit IgG labeled with horseradish peroxidase. The expression of P21 and COL1A1 was visualized using 3,3′‐diaminobenzidine tetrahydrochloride (DAB‐4HCl).

### 2.9 Transient RNA Interference and POT1 Overexpression Vector Transfection

Three specific short interfering RNA (siRNA) sequences were chemically synthesized by GenePharma (Shanghai, China) to target human POT1. The sequences for the POT1 siRNAs are as follows:

- *si‐POT1‐1:5′‐CCAGAUGUCAAGCUACAAA‐3′*.
- *si‐POT1‐2:5′‐GAAAGAUGUCAACAGCUAU‐3′*.
- *si‐POT1‐3:5′‐GGCUGAAGAUUCAAGUAUA‐3′*.

For the non‐targeting control siRNA (NC), a scrambled sequence of 5′‐UUCUCCGAACGUGUCACGUTT‐3′ was utilized. Cells were transiently transfected using Lipofectamine RNAiMax (Invitrogen) with 100 pmol of the respective siRNA sequences. The three specific sequences were mixed for POT1 interference, and after a 72‐h transfection period, cells were harvested for subsequent experiments.

### 2.10 Immunofluorescence

The lung specimens were fixed by immersing them in a 4% paraformaldehyde solution. As previously described, pHLAT2 cells were seeded in confocal dishes and transfected with POT1 siRNA. Then, the lung tissues or cells were subjected to staining using the following antibodies: anti‐P21 (Abclonal, A22460), anti‐S‐PC (Abcam, Cambridge, UK), and anti‐DNMT3A antibody (Abcam, A2065). FITC‐conjugated anti‐mouse IgG or Alexa Fluor 594‐conjugated anti‐rabbit IgG (Sigma, USA) were used to visualize the stained specimens. Additionally, the nuclei were stained with 4′,6‐diamidino‐2‐phenylindole (DAPI).

### 2.11 Cell Viability Assay

Cell viability was determined using the Cell Counting Kit‐8 (CCK‐8) kit from Dojindo Laboratory (Kumamoto, Japan), following the manufacturer's protocol. Briefly, 1000–2000 cells transfected with *POT1* siRNA or negative control (NC) were seeded into individual wells of a 96‐well plate. After incubation, each well added 10 μL of CCK‐8 reagent to 90 μL of culture media. Subsequently, the cells were incubated for 1 h at 37°C. The absorbance of the samples was measured at 450 nm using an MRX Microplate Reader (Dynex Technologies, West Sussex, UK) to determine the level of cell viability.

### 2.12 Wound Healing

Fibroblasts were cultured in DMEM with 0.1% FBS added to prevent cell proliferation, and the cultures were allowed to reach confluence before performing scratch assays. The extent of wound healing was determined by measuring the maximal distance between the scratch edges. The migration rate was evaluated by calculating wound repair using the equation: wound repair = 100 × (A—B)/A, in which A represents the initial width of the scratch (0 h), and B indicates the width after 24 or 48 h.

### 2.13 Cell Cycle Assay

The distribution of cell cycle phases was evaluated using flow cytometry with propidium iodide (PI) staining. Following the instructions from the Cell Cycle Analysis Kit (Beyotime, Shanghai, China), cells were treated with TGF‐β1, CSE, and PBS for 72 h in 6‐well plates. The cells were then collected, fixed in 70% ethanol at 4°C overnight, and stained with a PI/RNase A mixture. After incubation in the dark at 37°C for 30 min, the stained cells were analyzed with a flow cytometer (FC500 Flow Cytometer; Beckman Coulter, USA).

### 2.14 SA‐β‐Gal and ROS Staining

SA‐β‐Gal and ROS were detected according to the manufacturer's protocol of Servicebio (G1073‐100T) and Beyotime (S0033S), respectively.

### 2.15 Clonal Bisulfite Sequencing

The Qiagen EpiTect Bisulfite Kit was used to bisulfite the genomic DNA, followed by clonal bisulfite sequencing. Qiagen Quickspin columns were used to purify the bisulfite‐treated DNA. The bisulfite‐treated DNA was then amplified by PCR to obtain the 5′‐UTR section of POT1 containing a CpG island (criteria used: island size > 100, GC percentage > 50.0, Obs/Exp > 0.6). The amplified fragments were then cloned into Takara's pMD19‐T vector. Twenty clones were randomly selected from the PCR results, and their CpG island methylation levels were determined by sequencing. The primers used for the bisulfite‐PCR were as follows:

- forward primer, 5′‐GGGGTTTGTATTTTGGTTTTG‐3′,
- and reverse primer, 5′‐ACTCAAATAAACACACCAAACCTT‐3′.

### 2.16 ELISA Analysis

The protein levels in the human and mouse samples were measured using the ELISA kits (R&D Systems, Minneapolis, MN). The protocol followed the manufacturer's instructions.

### 2.17 Flow Cytometry for Primary Cell Sorting

The human or mouse lungs were freshly diced and rinsed with basic DMEM. Subsequently, the tissues underwent digestion in a mixture consisting of 2 mg/mL collagenase I, 1 mg/mL DNase I, 0.05 mg/mL dispase, and 0.04 mg/mL elastase. This digestion process occurred at 37°C in a water bath for 45 min. FBS was added to halt the digestion and achieve a final concentration of 10%. The resulting cell suspension was filtered through a 100 mm cell strainer (Miltenyi Biotec, Germany) to eliminate undigested tissues. After a 10‐min incubation with red blood cell lysate, the cell suspension was further filtered through 70 mm and 40 mm cell strainers to remove any remaining multicellular debris. Next, the cells were centrifuged at 500 × g at 4°C for 10 min, washed once in PBS, and finally resuspended in 2% FACS buffer. Primary cells were sorted and verified as in previous studies (Shi et al. 2022). FACS sorting was conducted using a BD FACS Aria II (BD Biosciences) for 30 min on ice. After incubation, the cells were washed, resuspended in 2% FACS buffer, and analyzed by flow cytometry. FAC‐sorted endothelial cells, epithelial cells, and fibroblasts were collected and subjected to mRNA extraction and qPCR analysis.

### 2.18 Construction of Luciferase Reporter Plasmids Containing *POT1* CpG Island Transfection and Luciferase Assays

A fragment measuring 219 base pairs from the human *POT1* CpG island was synthesized directly and subsequently cloned into the pGL3‐Basic luciferase vector (Promega). Afterward, these constructs were transiently transfected into cells utilizing Lipofectamine 3000 (Invitrogen), alongside co‐transfection with the Renilla pRL‐TK construct (Promega) for normalization purposes. Luciferase activity measurements were taken 24 h later using the Dual‐Luciferase Reporter Assay kit (Promega) on a TD20/20 Luminometer (Turner Designs). Each experiment was conducted in triplicate. The results are presented as relative luciferase activities, normalizing against Renilla luciferase activities.

### 2.19 Chromatin Immunoprecipitation (ChIP) Assay

The EZ‐ChIP Chromatin Immunoprecipitation kit (Upstate) was utilized to conduct a ChIP assay. Cells cultured in 15‐cm dishes were fixed using 1% formaldehyde for 10 min at room temperature. The cross‐linked cells underwent two washes with phosphate‐buffered saline (PBS) before being scraped in PBS supplemented with protease inhibitors. Cell nuclei were isolated by centrifugation at 700 × *g* for a duration of 4°C and then resuspended in SDS lysis buffer containing protease inhibitors. The mixture was sonicated on ice, resulting in the shearing of cross‐linked DNA to sizes ranging from 200 to 1000 base pairs. Subsequently, insoluble material was eliminated through centrifugation at 13,000 × *g* for 10 min. A volume of 100 μL from the supernatant was diluted in 900 μL of buffer with protease inhibitors and was then incubated with Protein G Agarose for 1 h at 4°C. After a brief centrifugation, 10 μL (1%) of the supernatant was reserved as the Input at 4°C, while the remainder was allocated for immunoprecipitation, where it was incubated with 5 μg of anti‐MECP2 and anti‐FOXP2 antibodies overnight at 4°C. A rabbit IgG antibody served as a negative control. The antibody/chromatin complexes were then incubated with Protein G Agarose for an additional hour at 4°C, and the resulting Protein G Agarose‐antibody/chromatin complex was harvested through brief centrifugation. After washing and centrifugation, the complex and Input were eluted to isolate protein‐DNA complexes. De‐cross‐linking occurred to liberate DNA by incubating in NaCl, RNase A, EDTA, and proteinase K solution, followed by DNA purification using spin columns. The ChIP DNA fragments were subjected to PCR, which targeted amplifying the *POT1* CpG island sequence containing the FOXP2 binding site (5′‐TGTGTTTATTT‐3′). The specific primary primers utilized for PCR amplification were: 5′‐AGGCGTTATTAGGTGTGAATCGT‐3′ (forward) and 5′‐ACTCAAATAAACACACCAAACCTT‐3′ (reverse).

### 2.20 Co‐Immunoprecipitation

The Co‐Immunoprecipitation (Co‐IP) was performed using an IP/Co‐IP Kit obtained from Thermo Fisher Scientific. In brief, 100 μL of antibody binding and washing buffer was used to wash the Protein A/G‐Agarose beads. The Protein A/G‐Agarose beads were then collected and gently rotated with rabbit anti‐MECP2 and anti‐FOXP2 antibodies for 10 min. The antibody‐bead complex was then incubated with 400 μg of total protein extracted using hypotonic lysis buffer for an additional 5 min. After removing the supernatant, the antibody‐protein‐bead complex underwent three washes with the washing buffer. Finally, the proteins bound to the antibody were eluted from the bead complex by gently resuspending in 100 μL of elution buffer for 2 min. The resulting protein samples were then analyzed using Western blot techniques.

### 2.21 Quantification Analysis

RNA sequencing. Following the manufacturer's instructions, total RNA was extracted from mouse lungs, and cDNA libraries were prepared using a KAPA RNA HyperPrep Kit (Kapa Biosystems, Wilmington, MA, USA). The cDNA libraries were sequenced using an Illumina HiSeq X Ten system (Illumina, USA). Kallisto and Deseq2 were used to analyze the transcriptome data.

### 2.22 Mouse Lung Tissue Samples and Single‐Nucleus RNA Sequencing

Cryopreserved mouse lung tissues from control (CTR) mice and mice subjected to bleomycin‐ and CS‐induced pulmonary fibrosis were used for single‐nucleus RNA sequencing (snRNA‐seq). Nuclei isolation was performed from frozen lung tissues using mechanical homogenization followed by gradient centrifugation‐based purification. Single‐nucleus libraries were constructed using the 10× Genomics Chromium Single‐Cell 3′ Gene Expression kit according to the manufacturer's instructions. Sequencing was performed on an Illumina NovaSeq instrument. Raw sequence data were aligned to the mouse genome (mm10 reference) using Cell Ranger software (v6.1.1). Nuclei with fewer than 400 unique molecular identifiers (UMIs), more than 10% mitochondrial transcripts, or predicted doublets (DoubletFinder, v2.0.3, recommended parameters) were excluded from further analysis.

### 2.23 Human Lung Tissue Samples and Single‐Cell RNA Sequencing

Fresh human lung tissues from healthy control donors (DONOR) and patients with pulmonary fibrosis (PF) underwent single‐cell RNA sequencing (scRNA‐seq). Single‐cell suspensions were prepared by enzymatic and mechanical dissociation. Libraries were generated using the 10× Genomics Chromium platform and sequenced on an Illumina NovaSeq system. Raw sequencing data were aligned to the GRCh38 human reference genome using Cell Ranger (v6.1.1). Quality filtering (removal of cells with fewer than 500 transcripts, more than 15% mitochondrial genes, and predicted doublets) followed a similar protocol described for mouse tissues.

### 2.24 Data Processing and Clustering

Standard data integration workflow from the Seurat package (v4.3.0) was applied to integrate and harmonize datasets across samples, accounting for potential batch effects. Briefly, the 5000 most variable genes identified using Seurat's FindVariableFeatures function (selection.method = “vst”) were used for integration. Principal Component Analysis (PCA) was performed based on these variable features, and the top 20 (human) and 16 (mice) principal components were selected for subsequent unsupervised clustering and visualization. Batch‐corrected clustering was conducted using the Louvain algorithm with a resolution of 0.4 (human) and 0.3 (mice) to identify distinct cellular populations. Uniform Manifold Approximation and Projection (UMAP) based on these principal components was employed for the visualization of cellular clusters. Cell identities were determined through expression profiling of canonical marker genes referenced from established literature.

### 2.25 Cellular Senescence Scoring

Cellular senescence scores for each cell were computed using Seurat's AddModuleScore function. The senescence‐related gene signature utilized for scoring was derived from the Aging Atlas database. Senescence scores were compared across DONOR/CTR, non‐smoking PF, and smoking PF groups, visualized by violin plots, and further evaluated at the individual cell‐type level to explore cell type‐specific senescence responses.

### 2.26 Statistical Analysis

The associations of smoking and fibrosis‐related RNAs (i.e., *CDKN1A*, *CDKN2A*, and *TP53*) with PF status were estimated using logistic regression. The associations between smoking and fibrosis‐related RNAs were calculated using linear regression using a gene expression dataset (GSE47460, including 122 patients with IPF and 92 controls) from the Gene Expression Omnibus (GEO, http://www.ncbi.nlm.nih.gov/geo). Age, sex, and BMI were included in the regression models as covariates. The gene expression data was log‐transformed and z‐scored before regression. Gene set analysis was carried out in R using the clusterProfiler package (version 4.4.4) to identify up or downregulated gene sets according to Gene Ontology (GO) terms and KEGG pathways (Wu et al. 2021). Multiple comparison tests and one‐ or two‐way Analysis of Variance (ANOVA) were employed to examine the impact of CS on cell senescence and PF with Bleo or TGF‐β1 activation. One‐way ANOVA detected methyltransferases, POT1, and senescent cell markers in FAC‐sorted primary cell types. Two‐sided Student's *t*‐tests were used to assess POT1's therapeutic impact. Dunnett's and Tukey's tests were used appropriately to compare multiple treatment groups, multiple pairings of control and treatment, or multiple treatments and single control. *p* values were deemed significant if they were less than 0.05. All data analyses were conducted in R (version 4.3.2).

## 3 Result

### 3.1 Cigarettes Exacerbate Bleo‐Induced Pulmonary Fibrosis by Increasing Cell Senescence

**FIGURE 1:**
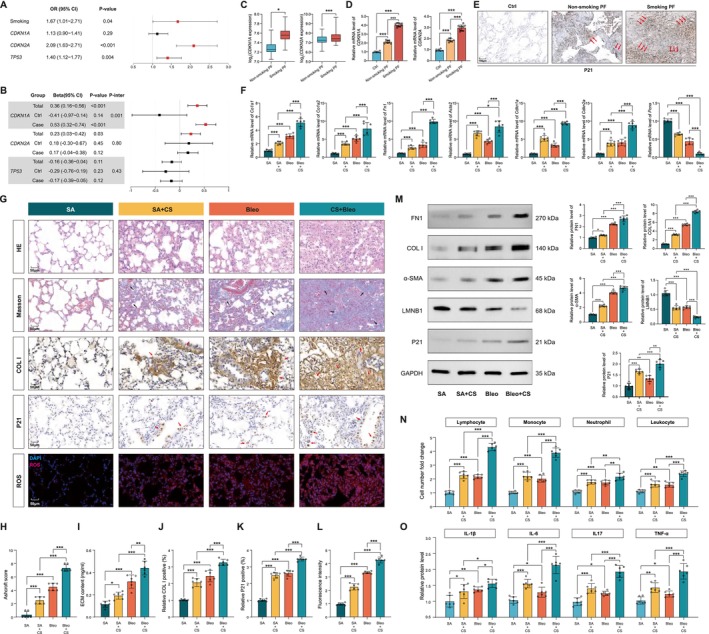
Cell senescence is involved in smoking‐induced PF. (A) The association between smoking and PF and between senescent markers *CDKN1A, CDKN2A, TP53*, and PF. The odds ratios (OR) were calculated using logistic regression, with adjustments of age, sex, and BMI. (B) The association between smoking and senescent markers *CDKN1A, CDKN2A, TP53*. The β coefficients were calculated using linear regression, with adjustments of age, sex, and BMI. (C) The mRNA levels of *CDKN1A and CDKN2A* in PF lungs with or without smoking. (D) The mRNA levels of *CDKN1A and CDKN2A* in Ctrl (*N* = 6) and PF lungs with (*N* = 10) or without smoking (*N* = 8). (E) IHC analysis of P21 protein. (F) The mRNA levels of fibrotic and senescent cell genes. (G) HE and Masson staining of mouse lungs, and IHC assay of COL I, P21, and ROS in mouse lungs. Scale bar: 50 μm. (H) The Ashcroft score was calculated. (I) Soluble collagen contents in lung homogenate. (J–L) Quantification of COL I, P21, and ROS in mouse lungs. (M−M1) Western blot analysis of fibrosis‐ and senescent‐related proteins in mouse lungs. (N) Cell count in BALF. (O) ELISA analysis of inflammatory cytokines in BALF. *N* = 6 per group in data D–O. Data in A–C were generated from GSE47460. Data in C were evaluated using the Student's *t*‐test. Data in D were evaluated by one‐way ANOVA (Tukey test). Data in F, H–L, and M1–O were evaluated by two‐way ANOVA (Tukey test). *p < 0.05, **p < 0.01, ***p < 0.001 versus control. Data are presented as the mean ± SEM.

By analyzing the public database GSE47460, which contained 92 normal controls and 122 idiopathic pulmonary fibrosis/unusual interstitial pneumonia (IPF/UIP) patients, we found that compared with non‐smokers, individuals with a history of smoking had higher odds of IPF (OR = 1.67, 95% CI = 1.01–2.71, *p* = 0.04). Additionally, the increased expression of *the CDKN2A* gene is significantly associated with the increased presence of IPF (OR = 2.09, 95% CI = 1.63–2.71, *p* < 0.001), as is the TP53 elevation (OR = 1.40, 95% CI = 1.12–1.77, *p* = 0.003). The association between the *CDKN1A* expression and IPF also showed a risk trend, though it did not reach statistical significance (OR = 1.13, 95% CI = 0.9–1.41, *p* = 0.29) (Figure 1A). In the total population, smoking is associated with elevated expression of *CDKN1A* (*β* = 0.36, 95% CI = 0.16–0.56, *p* = 3.69 × 10^−4^) and *CDKN2A* (*β* = 0.23, 95% CI = 0.03–0.42, *p* = 2.57 × 10^−2^). Notably, the association between smoking and gene expression is modified by IPF status. For example, smoking is significantly associated with increased *CDKN1A* expression in individuals with IPF (*β* = 0.53, 95% CI = 0.32–0.74, *p* < 0.001), while this association is diminished in non‐PF individuals (*β* = −0.41, 95% CI = −0.97–0.14, *p* = 0.14). These findings suggest that smoking may influence the development of IPF by promoting the expression of senescence‐related genes (Figure 1B). Further analyses revealed significantly increased expression of *CDKN1A* and *CDKN2A* in smoking IPF patients compared to non‐smoking IPF patients (Figure 1C). We next validated that CDKN1A and CDKN2A were progressively higher in normal, non‐smoking IPF patients and smoking IPF patients by qPCR and immunohistochemistry (IHC) analysis (Figures 1D,E and S1A).

Next, C57/BL6 mice were exposed intratracheally to Bleo and/or cigarette inhalation to establish mouse PF (Figure S1B). qPCR assay showed that both Bleo and cigarette alone significantly induced PF, as supported by increased fibrotic genes *Col1a1, Col1a2, Fn1*, and *Acta2* (Figure 1F). Moreover, these fibrotic genes were further expanded in the cigarette combined with Bleo groups. Interestingly, the senescent cell markers cyclin‐dependent kinase inhibitor 1a (*Cdkn1a*, also known as P21) and cyclin‐dependent kinase inhibitor 2a (*Cdkn2a*, also known as p16) were significantly increased. In contrast, the proliferative marker *Pcna* was significantly decreased in the Bleo and cigarette groups, and these trends were further amplified by Bleo‐combined cigarette treatments (Figure 1F). Histological staining and western blotting experiments were performed to further verify cell senescence involved in cigarettes‐ and Bleo‐induced PF. H&E (Hematoxylin, eosin), Masson's trichrome staining, and Ashcroft scores showed that the injured and fibrotic areas were significantly increased after cigarette and Bleo treatment, compared to control mice, and that these levels were further augmented in cigarettes‐exposed Bleo‐treated mice (Figure 1G−H). Consistently, IHC staining and Sircol assay showed that the levels of fibrotic proteins COL1A1 and Extracellular matrix (ECM) content were highest in cigarette‐exposed Bleo‐treated mice compared to either cigarette or Bleo alone groups (Figure 1G,I,J). Consistent with the degree of fibrosis, the highest degree of cell senescence was observed in the combination groups compared to other groups, as supported by senescent markers detection, including increased P21 and ROS generation (Figure 1G,K,L). Western blot analysis further demonstrated cell senescence was accompanied by fibrosis responses in PF mouse lungs (Figure 1M). Cell count and ELISA detection of Bronchoalveolar Lavage Fluid (BALF) revealed enhanced inflammation cell infiltration and inflammatory cytokines secretion in CS and Bleo groups, and highest in combination groups (Figure 1N−O).

These results suggested cigarettes induced significant PF and cell senescence, which may confer the ability of cigarettes to exacerbate Bleo‐induced PF.

### 3.2 Cigarettes Induced Type II Alveolar Cell Senescence In Vivo and In Vitro

**FIGURE 2:**
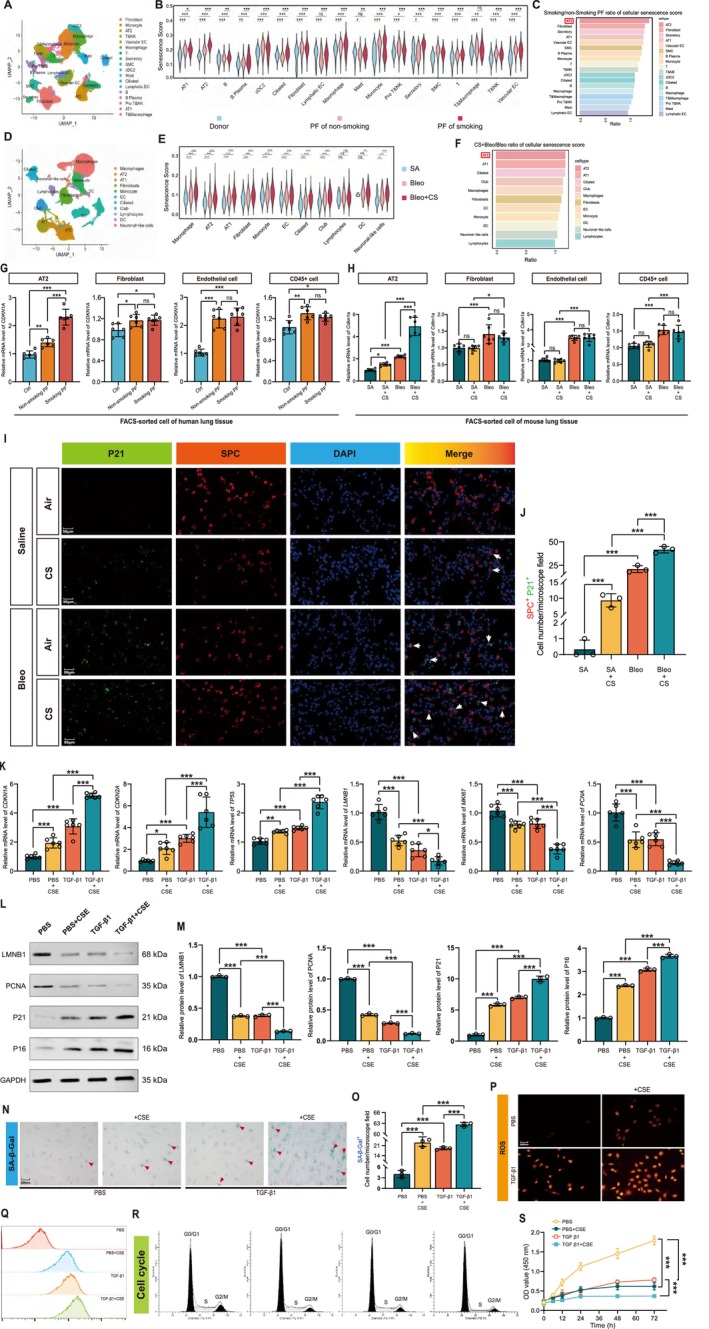
Excessive cell senescence of AT2 cells in PF patients and mice, and CSE induced and augmented TGF‐β‐mediated senescence in pHLAT2 cells. (A) UMAP visualization displaying annotated lung cell populations identified in DONOR and PF groups. (B) Cellular senescence scores for each identified cell type among DONOR, non‐smoking PF, and smoking PF groups. (C) The ratio of cellular senescence scores (smoking PF/non‐smoking PF) for individual cell types. (D) UMAP visualization displaying annotated mouse lung cell populations identified in different groups. (E) Cellular senescence scores for each identified cell type among SA, Bleo, and Bleo + CS groups. (F) The ratio of cellular senescence scores (Bleo + CS/Bleo PF) for individual cell types. (G) The mRNA levels of senescent marker *CDKN1A* in sorted primary AT2, fibroblast, endothelial, and CD45‐positive immune cells of PF patients. *N* = 6 per group. (H) The mRNA levels of senescent marker *Cdkn1a* in sorted primary AT2, fibroblast, endothelial, and CD45‐positive immune cells of PF mice. *N* = 6 per group (I,J) Double immunofluorescence detection and cell count of P21^+^ SP‐C^+^ in mouse lungs. Scale bar: 50 μm. *N* = 3 per group. (K) qPCR detection of cell senescence markers. (L,M) Western blot of cell senescence‐related proteins. (N,O) SA‐β‐gal staining. (P,Q) Immunofluorescence detection of ROS by cellular immunofluorescence observations and flow cytometry analysis. (R,S) Cell cycle and viability detection. All data were evaluated by two‐way ANOVA (Tukey test). Data in B was evaluated by one‐way ANOVA (Tukey test). Two‐way ANOVA (Tukey test) evaluated data in C, E, and K**–**S. *p < 0.05, **p < 0.01, ***p < 0.001 versus control. Data are presented as the mean ± SEM. All experiments were repeated three dependent times.

To identify the cell types responsible for cell senescence in cigarettes exacerbated PF lungs, we performed scRNA‐seq and snRNA‐seq analyses in human PF patients and PF mice, respectively. For scRNA‐seq analysis of human PF patients, we collected the lung tissues from 3 healthy donors, 3 PF patients without smoking, and 3 PF patients with smoking. We finally obtained 129,162 high‐quality cells through data quality control, including 30,657 cells from donor lung tissues, 46,590 from non‐smoking PF lung tissues, and 51,915 cells from smoking PF lung tissues. The UMAP results showed that the lungs could be divided into 18 types of cell populations, such as alveolar epithelial cells, vascular endothelial cells, fibroblasts, immune cells, and so on (Figure 2A). We next found that the cellular senescence scores were gradient increased from donor to non‐smoking PF patients and to smoking PF patients, along with a gradient increase of fibrotic gene levels (Figure S2A,B). The gene panel used to evaluate the senescence scores was constructed using the aging cell atlas. Further analysis revealed that the cellular senescence scores were significantly increased in almost all cell types, including AT1, AT2, fibroblast, vascular endothelial cells, macrophage, and other cell types from both non‐smoking and smoking PF patients compared to donors (Figure 2B). Moreover, the fold change of cellular senescence scores was highest in AT2 cells between non‐smoking PF patients and smoking PF patients (Figure 2C). Subsequently, we performed snRNA‐seq analysis of mice with Sa, BLM, and BLM + CS treatments (*N* = 3 per group). We finally obtained 69,383 high‐quality cells through data quality control, including 10,782 cells derived from Sa lung tissues, 25,900 cells derived from BLM lung tissues, and 32,701 cells derived from BLM + CS lung tissues. The UMAP results showed that the lungs could be divided into 11 types of cell populations, such as alveolar epithelial cells, vascular endothelial cells, fibroblasts, immune cells, and so on (Figure 2D). Moreover, the cellular senescence scores were widely increased in mouse lungs exposed to BLM or both smoking, and the fold change of cellular senescence scores was highest in AT2 cells between non‐smoking PF mice and smoking PF mice, similar to the results observed in PF patients (Figures 2E,F and S2C,D).

Next, we sorted primary endothelial, AT2, fibroblast, and CD45‐positive immune cells by FACS sorting, and it was found that the mRNA levels of CDKN1A in AT2 cells moderately increased in PF patients without smoking and further increased in smoking PF patients (Figure 2G). Although *CDKN1A* levels also increased in PF patients' endothelial, fibroblast, and CD45‐positive cells, smoking failed to further increase *CDKN1A* expression in these cells (Figure 2G). Similar results were also observed in mouse PF lungs (Figure 2H). Furthermore, the co‐localization of P21‐positive cells with SP‐C‐positive cells further indicated that cigarettes exacerbated PF lung senescence, mainly originating from AT2 cells (Figure 2I,J). It appeared that AT2 was one of the most sensitive cell types in response to cigarettes exacerbating PF lung senescence. Next, we measured telomere length in AT2 cells from smoking‐exposed and control mice using qFISH. We found that the length of the telomere was significantly shortened in BLM groups and further shortened in smoking‐exposed BLM groups, suggesting CS‐induced senescence links to telomere shortening (Figure S2E). These results suggest that cigarettes reinforce the senescence of AT2 cells to exacerbate Bleo‐induced PF.

TGF‐β1‐mediated activation of the TGF‐β/SMAD signaling pathway is essential in promoting alveolar tissue damage and fibrogenesis during PF. We next used sorted primary human AT2 cells (pHLAT2) to investigate the effects of TGF‐β1 and cigarettes on cell senescence. As shown in Figure 2K, TGF‐β1 increased *CDKN1A*, *CDKN2A*, and *TP53* while decreasing *LMNB1, MKI67*, and *PCNA* at mRNA levels. Similar results were found in cigarette smoke extract (CSE)‐treated pHLAT2 cells. Moreover, CSE exposure further enhanced the TGF‐β1‐induced upregulation of *CDKN1A* and *CDKN2A* and downregulation of *LMNB1, MKI67*and *PCNA*. Western blot revealed CSE augmented TGF‐β1‐induced senescence (Figure 2L,M). SA‐β‐gal staining and quantitation of SA‐β‐gal^+^ cells revealed cigarettes could strikingly boost TGF‐β1‐induced cell senescence (Figure 2N,O). In addition, ROS levels were higher in the TGF‐β1 and cigarette groups and highest in the combination groups (Figures 2P,Q and S2F,G). In contrast, cell viability and cell cycle were reduced by TGF‐β1 and cigarette, especially in the combination groups (Figures 2R,S and S2H).

Our in vivo and in vitro experiments have consistently demonstrated that cigarettes induce AT2 cell senescence and may reveal the underlying mechanism for cigarette‐induced PF.

### 3.3 Cigarettes Induce Senescence by Downregulating POT1

**FIGURE 3:**
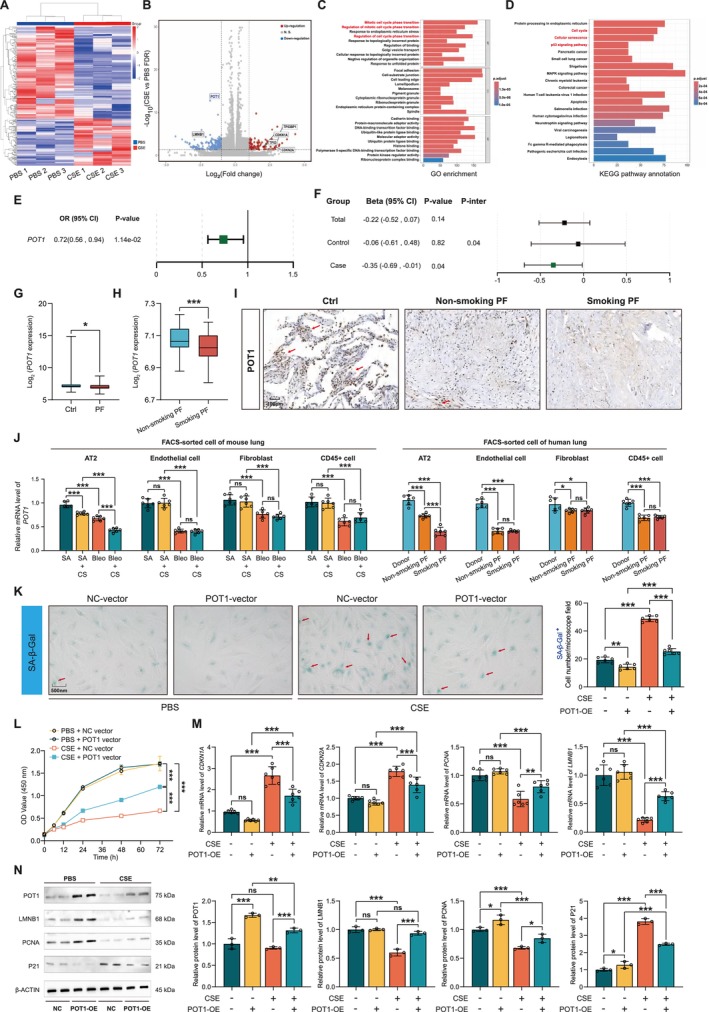
CSE induced and augmented TGF‐β‐mediated senescence in pHLAT2 cells. (A,B) The heatmap and volcano diagram of DEGs between CSE‐ and PBS‐treated pHLAT2 cells. (C,D) GO and KEGG orthologs of the upregulated and downregulated DEGs with CSE treatment. (E) The association between *POT1* levels and PF. The odds ratios (OR) were estimated using logistic regression, with adjustments for age, sex, and BMI. (F) The association between smoking and *POT1* levels. The β coefficients were calculated using linear regression, with adjustments for age, sex, and BMI. (G,H) The mRNA levels of *POT1* in PF lungs with or without smoking. (I) IHC analysis of POT1 protein. (J) The mRNA levels of *POT1* in FAC‐sorted primary cells from PF mice and patients. *N* = 6 per group. (K) SA‐β‐gal staining after POT1 overexpression with or without CSE treatment in pHLAT2 cells. (L) Cell viability in different groups. (M,N) qPCR and western blot analysis of cell senescence‐related proteins. Data in E – H were generated from GSE47460. Data in G and H were evaluated using the Student's t‐test. Data in J – N were evaluated by two‐way ANOVA (Tukey test). *p < 0.05, **p < 0.01, ***p < 0.001 versus control. Data are presented as the mean ± SEM. All experiments were repeated three dependent times.

We then performed RNA‐seq analysis to explore the transcriptomic changes toward cigarette stimulation and found that cigarettes induced a distinct RNA profile (Figure 3A). The volcano plot showed that the senescence markers *CDKN1A*, *CDKN2A*, *TP53*, and *TP53BP1* were significantly increased, while *LMNB1* was decreased by cigarettes, further supporting our finding that cigarettes have a promoting role in cell senescence. We found that *POT1* was one of the most downregulated differentially expressed genes (DEGs) (Figure 3B). Enrichment analysis showed that genes involved in the cell cycle, cellular senescence, and p53 signaling pathway were significantly upregulated, whereas genes involved in cell cycle phase transition were downregulated (Figure 3C,D). Analysis using the GSE47460 database revealed a protective effect of POT1 against IPF (OR = 0.72, 95% CI, 0.56–0.94, *p* = 0.014) (Figure 3E). Moreover, smoking is associated with decreased expression of *POT1* in IPF patients (*β* = −0.35, 95% CI = −0.69 to −0.01, *p* = 0.0419) (Figure 3F). Consistently, the mRNA levels of *POT1* were decreased in IPF compared to normal controls (Figure 3G), and smoking IPF patients harbored a lower *POT1* level than non‐smoking IPF patients (Figure 3H). IHC further revealed that POT1 was progressively lower in normal, non‐smoking IPF patients and smoking IPF patients (Figures 3I and S3). We further examined POT1 expression in FAC‐sorted primary endothelial, AT2, fibroblast, and immune cells and found that *POT1* was significantly reduced in all four types of cells, both in patients with PF and in mouse models. However, only in AT2 cells, *POT1* was further reduced by smoking (Figure 3J). To investigate the role of POT1 in cigarette‐induced senescence, POT1 was inductively overexpressed in pHLAT2 cells. SA‐β‐gal staining demonstrated that POT1 inhibited CSE‐induced cell senescence (Figure 3K). Additionally, CCK8 revealed that POT1 effectively alleviated CSE‐induced cell growth inhibition (Figure 3L). qPCR and western blot consistently revealed POT1 suppressed cigarette‐induced elevation of P21 and reduction of LMNB1 and PCNA (Figure 3M,N).

### 3.4 Cigarettes Downregulate POT1 via Synergetic Inducing MECP2‐Mediated DNA Methylation and Inhibition of FOXP2 Binding

**FIGURE 4:**
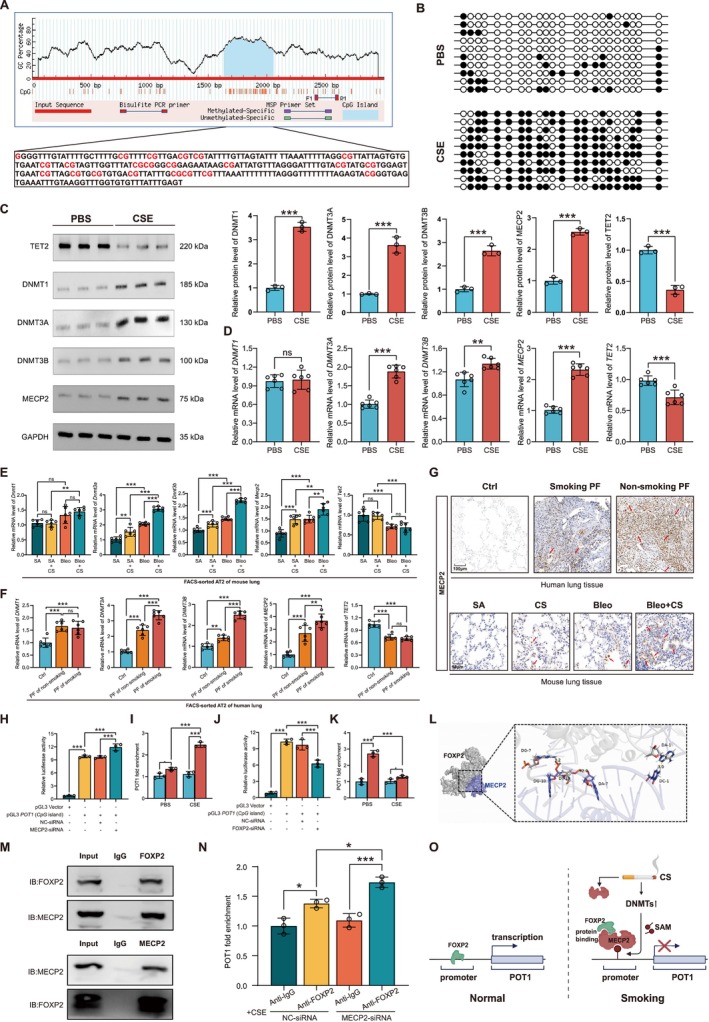
CSE‐induced hypermethylation of POT1 CpG island and induced a MECP2‐FOXP2 complex to inhibit *POT1* transcription. (A) The DNA sequence of CpG island in POT1 promoter. (B) The methylation levels of every CpG site in *POT1* CpG island with CSE stimulation in pHLAT2 cells. (C) The protein expression of methyltransferases and demethyltransferases after CSE treatment in pHLAT2 cells. (D) The mRNA expression of methyltransferases and demethyltransferases after CSE treatment in pHLAT2 cells. (E) The mRNA expression of methyltransferases and demethyltransferases in AT2 cells of CS‐treated mice. (F) The mRNA expression of methyltransferases and demethyltransferases in AT2 cells of PF patients. (G) IHC examination of MECP2 levels in PF patients and mice with or without smoking. (H) Dual‐luciferase reporter gene analysis of the activity of *POT1* CpG with *si‐MECP2* treatment. (I) ChIP‐qPCR analysis of the activity of *POT1* CpG with MECP2 antibody. (J) Dual‐luciferase reporter gene analysis of the activity of *POT1* CpG with *si‐FOXP2* treatment. (K) ChIP analysis of the activity of *POT1* CpG with FOXP2 antibody. (L) *In silico* prediction of the MECP2 – FOXP2 interaction by molecular docking. (M) Co‐IP analysis with MECP2 and FOXP2 antibodies. (N) ChIP analysis of the activity of *POT1* CpG with FOXP2 antibody in CSE‐treated AT2 cells with *si‐MECP2* treatment. (O) Schematic diagram of the synergistic effect of methylation and MECP2 on POT1 transcriptional inhibition. Data in C were evaluated using the Student's *t*‐test. Data in E, H, I, and N were evaluated by two‐way ANOVA (Tukey test). Data in F were evaluated by one‐way ANOVA (Tukey test). *p < 0.05, **p < 0.01, ***p < 0.001 versus control. Data are presented as the mean ± SEM. All experiments were repeated three dependent times.

One question is how cigarettes cause the downregulation of POT1. We first analyzed the potential CpG island in the promoter of POT1, and a CpG island was found at the 11,639–2057 bp upstream of the *POT1* transcription start site (Figure 4A). DNA methylation detection based on the Bisulfite sequencing PCR (BSP)‐method revealed CSE‐induced significant upregulation of methylation levels of the *POT1* DNA promoter (Figures 4B and S4A). Subsequently, western blot and qPCR analysis consistently revealed the amount of DNA methylation‐related genes was significantly altered after CSE treatment, including increased DNMT1, DNMT3A, DNMT3B, and MECP2, while TET2 was decreased (Figure 4C,D). Consistently, 5‐Azacytidine (5‐Aza), an inhibitor of DNA methylation, significantly restored CSE‐induced POT1 downregulation both at mRNA and protein levels (Figure S4B,C). To further confirm whether aberrant methylation is involved in smoking‐exacerbated PF, we examined methyltransferase expression levels in FAC‐sorted primary endothelial, AT2, fibroblast, and immune cells. Results revealed that methyltransferases were significantly overexpressed in Bleo‐induced mouse PF, and *Dnmt3a, Dnmt3b*, and *Mecp2* were further upregulated in smoking mice (Figure 4E). In IPF patients, we consistently found that methyltransferases were involved in pulmonary fibrosis and that *DNMT3A, DNMT3B*, and *MECP2* were further elevated in AT2 cells in IPF patients with smoking (Figure 4F). Considering that MeCP2 can bind to methylated CpG through its methyl‐binding structural domain (MBD), thus acting as a transcriptional repressor, we next examined the protein expression of MECP2. IHC analysis further verified the increase of MECP2 in PF lungs and constantly increased with smoke exposure both in humans and mice (Figures 4G, and S4D,E).

Although we found that the *POT1* CpG island was hypermethylated, whether this CpG island is associated with reduced POT1 transcriptional expression remains unknown. Dual‐luciferase reporter gene detection assay showed the luciferase activity of the *POT1* CpG island‐containing PGL3‐Basic vector was significantly higher than that of the blank vector, suggesting that this DNA sequence of the *POT1* CpG island is involved in the transcriptional regulation of the POT1 gene. Besides, the luciferase activity of the *POT1* CpG island was decreased by CSE stimulation, which was blocked by 5‐Aza (Figure S4F). Moreover, the luciferase activity of the *POT1* CpG island was increased after MECP2 knockdown (Figure 4H). ChIP‐qPCR assay found an enrichment of *POT1* CpG with MECP2 antibody and was further enhanced by CSE treatment, suggesting MECP2 could target the *POT1* CpG island and inhibit POT1 transcription directly (Figure 4I). Besides, *in silico* prediction indicated FOXP2 was a potential POT1 transcription factor and that knockdown of FOXP2 significantly inhibited luciferase activity in the *POT1* CpG island region (Figure 4J). ChIP‐qPCR assay revealed an enrichment of *POT1* CpG with FOXP2 antibody but decreased by CSE treatment, suggesting MECP2 could target the *POT1* CpG island and, therefore, inhibit POT1 transcription directly (Figure 4K). MECP2 and FOXP2 exhibited a distinct opposite effect on POT1 transcription, thus introducing a question of how MECP2 and FOXP2 co‐regulate POT1 transcription. We performed molecular docking to elucidate this and found that MECP2 may interact with FOXP2 (Figure 4L). Co‐IP experiments showed that anti‐FOXP2 could effectively detect MECP2 protein, and similarly, anti‐MECP2 could effectively detect FOXP2 protein (Figure 4M). These experiments confirmed that FOXP2 and MECP2 proteins bind to each other. To investigate the effect of the FOXP2 – MECP2 complex on *POT1* CpG activity, we further examined the transcriptional activity of this region after MECP2 knockdown. ChIP‐qPCR experiments revealed that the knockdown of MECP2 significantly enhanced the enrichment of FOXP2 for the *POT1* CpG island (Figure 4N). To investigate whether DNMT3A and DNMT3B could affect FOXP2 activation, we examined the expression of FOXP2 after knockdown of *DNMT3A* and *DNMT3B*. It was found that neither knockdown of *DNMT3A* nor *DNMT3B* significantly altered the expression of FOXP2 (Figure S4G,H). Additionally, we detected the co‐localization of MECP2 and FOXP2 in human and mouse lung tissue and found that there was significant co‐localization of MECP2 and FOXP2 in AT2 cells of both human and mouse lung tissues (Figure S4I,J).

In conclusion, these results strongly suggest that MECP2 competes with FOXP2 for binding to the *POT1* CpG island and that high expression of MECP2 closes the binding of FOXP2 to the *POT1* CpG island. Smoking synergistically inhibits POT1 expression by inducing methylation of the *POT1* CpG island on the one hand and by inducing MECP2 expression to block transcription factor FOXP2 from binding to POT1 transcription on the other hand. The schematic diagram is shown in Figure 4O.

### 3.5 Knockdown of POT1‐Induced Epithelial Cell Senescence and Evoke Collagen Deposition of Fibroblast via SASP

**FIGURE 5:**
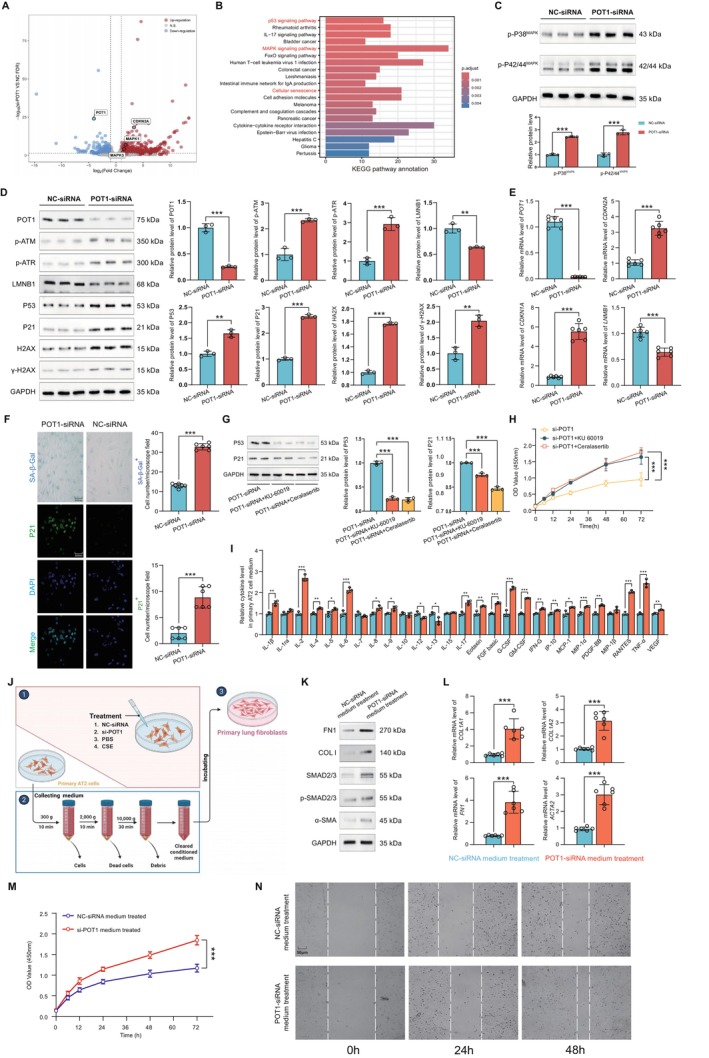
by activating ATM, ATR, P53, and H2AX pathways. (A,B) The volcano diagram and KEGG analysis of DEGs between NC siRNA‐ and *POT1* siRNA‐treated pHLAT2 cells. (C,D) The expression of p‐p38, p‐p42/44, senescent markers, and the activities of ATM, ATR, P53, and H2AX pathways after *si‐POT1*. (E) *si‐POT1* upregulated *CDKN1A* and *CDKN2A*, while downregulated *LMNB1*. (F) SA‐β‐gal and P21 staining after POT1 knockdown. (G) p‐ATM and p‐ATR inhibitors inhibited P21 and P53 expression. (H) p‐ATM and p‐ATR inhibitors elevated cell viability. (I) Reprogrammed SASP components after *si‐POT1*. (J–L) Fibrotic response in fibroblast after incubation with conditioned medium from NC‐ or *si‐POT1*‐treated AT2 cells. (M,N) Viability and migration of fibroblast cells after incubation with conditioned medium from NC‐ or *si‐POT1*‐treated AT2 cells. Data in A – C, F, I, and J were evaluated using the Student's *t*‐test. Data in D and E were evaluated by one‐way ANOVA (Dunnett's test). *p < 0.05, **p < 0.01, ***p < 0.001 versus control. Data are presented as the mean ± SEM. All experiments were repeated three dependent times.

As shown in Figure 5A,B, we examined the transcriptomic changes in AT2 cells after *POT1* knockdown. We found that the senescence pathway was significantly enriched in the upregulated DEGs, and MAPK was one of the most altered pathways after *POT1* knockdown. Western blotting further revealed that siRNA‐induced *POT1* knockdown increased P21 and activated the MAPK signaling pathway but decreased LMNB1 in pHLAT2 cells. Senescent regulatory pathways, including p‐ATM, p‐ATR, P53, and γ‐H2AX, were significantly increased after POT1 knockdown (Figure 5C,D). Accordingly, the mRNA levels of senescent markers *CDKN1A* and *CDKN2A* were increased, while *LMNB1* was decreased (Figure 5E). Furthermore, the number of senescent cells increased, as revealed by SA‐β‐gal and P21 staining (Figure 5F). Next, we found that both p‐ATM inhibitor KU‐60019 and p‐ATR inhibitor Ceralasertib suppressed P21 and P53 upregulation in *si‐POT1* groups, supporting that POT1 deficiency‐induced senescence was p‐ATM and p‐ATR dependent (Figure 5G). CCK8 assay revealed both KU‐60019 and Ceralasertib could elevate the cell proliferation rate in *si‐POT1* groups (Figure 5H).

To assess the effect of AT2 cell senescence on fibrosis, we prepared a conditional medium from epithelial cells pretreated with *si‐POT1* or NC. Multiplex cytokine analysis showed the amount of inflammatory and profibrotic factors was upregulated in the *POT1* siRNA groups compared to the NC groups (Figure 5I). Furthermore, conditional medium from *si‐POT1*‐pretreated AT2 cells significantly promoted the expression of the fibrotic proteins COL I, FN1, and ACTA2, as well as the activation of the TGF‐β/SMAD2/3 fibrotic pathway in fibroblasts (Figures 5J−L, and S5A). In addition, the proliferation and migration rate of fibroblasts was also accelerated by conditioned medium from *si‐POT1*‐pretreated AT2 cells (Figure 5M,N). To assess the effect of smoking‐exposed AT2 cell senescence on fibrosis, we prepared a conditional medium from CSE‐exposed AT2 cells. Western blot revealed that conditional medium from CSE‐exposed AT2 cells significantly promoted the expression of the fibrotic proteins COL I, FN1, and ACTA2 and the activation of the TGF‐β/SMAD2/3 fibrotic pathway in fibroblasts (Figure S5B,C).

These results suggest that POT1 deficiency may promote fibrosis by forcing senescent epithelial cells to secrete SASP components.

### 3.6 AAV9‐Mediated POT1 Restoration Released CS‐Induced PF In Vivo

**FIGURE 6:**
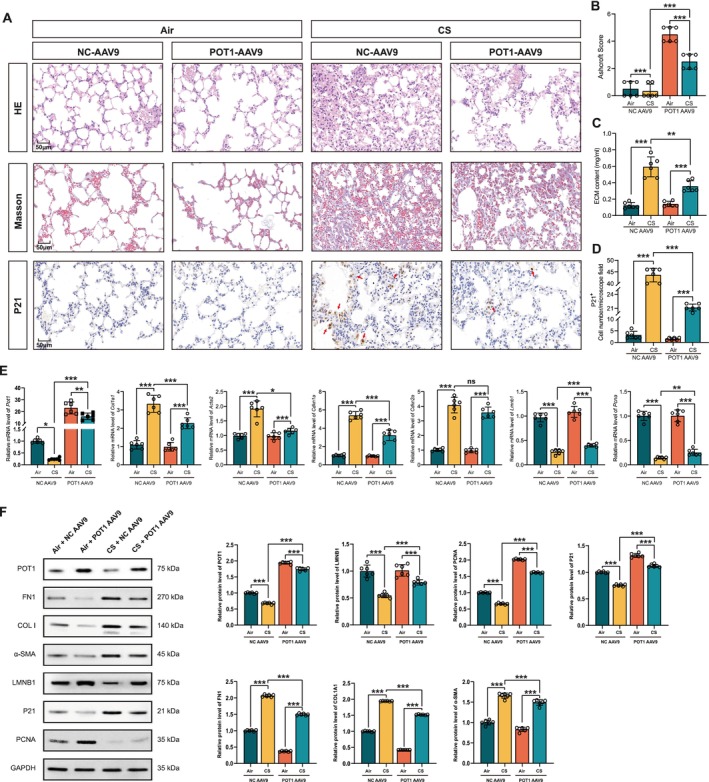
AAV9‐mediated POT1 overexpression alleviated PF in mice. (A) HE, Masson, and IHC staining of mouse lungs. (B–D) Ashcroft score, Sircol assay, and P21 staining of mouse lungs. (E) The transcriptional levels of *Pot1*, fibrotic genes, and senescence marker levels in mouse lungs. (F) AAV9‐POT1 suppressed fibrosis‐related proteins and senescent‐related proteins. *N* = 6 per group. All data were evaluated by two‐way ANOVA (Tukey test). *p < 0.05, **p < 0.01, ***p < 0.001 versus control. Data are presented as the mean ± SEM.

We designed AAV9 carrying POT1‐overexpressing vectors and control vectors (Figure S6A). The lungs of AAV‐POT1‐treated mice showed reduced lesion sizes and fibrotic areas compared to Bleo‐treated control mice and decreased P21 levels, as demonstrated by histological and IHC staining (Figure 6A). Additionally, the Ashcroft score assay, Sircol assay, and quantification of P21‐positive cell number were all decreased by AAV‐POT1 (Figure 6B−D). These results suggest a protective role of AAV‐POT1 against PF. After delivery of the AAV9‐POT1 to mouse lungs, upregulation of the *POT1* gene and protein was detected in both air and CS groups, and AAV‐POT1 delivery effectively restored CS‐induced endogenous POT1 reduction (Figure 6E). The fibrotic genes *Col1a1* and *Acta2* were significantly downregulated by AAV‐POT1 at mRNA levels (Figure 6E). As expected, the senescence markers *Cdkn1a* and *Cdkn2a* were downregulated, whereas *Lmnb1* and *Pcna* were rescued by POT1 (Figure 6E). Western blot further revealed AAV‐POT1 rescued CS‐induced POT1 downregulation in mouse lungs. Meanwhile, the fibrosis and senescence proteins were suppressed by POT1, as shown by decreased COL I, ACTA2, FN1, and P21 protein and increased LMNB1 and PCNA protein (Figure 6F). These results demonstrated that POT1 is associated with cell senescence in fibrotic lungs. In addition, we further examined the expression of POT1 in AT2 cells and found that POT1 protein was significantly elevated in sorted primary AT2 cells. These results suggest that AAV9‐POT1 can target at least AT2 cells in vivo (Figure S6B).

In conclusion, AAV9‐mediated overexpression of POT1 remarkably suppressed inflammation and fibrosis by blocking cell senescence in the mouse lung.

## 4 Discussion

Cellular senescence is thought to be involved in the onset and development of a wide range of diseases and is not just a physiological phenomenon of aging (Di Micco et al. 2021; Munoz‐Espin and Serrano 2014). The causes of cell senescence are diverse, including DNA damage, telomere shortening, oncogene activation, oxidative stress, epigenetic changes, and so on (Lopez‐Otin et al. 2023). In different diseases, the causes of senescence, the phenotypes, and the composition and role of SASP are di (Munoz‐Espin and Serrano 2014). There is a large number of cigarette smokers worldwide. Tobacco exposure is one of the major risk factors for PF (Bae et al. 2022). Smoking can not only cause PF spontaneously but also worsen the progression of other types of PF. For example, smoking exacerbates IPF, connective tissue disease‐associated PF (CTD‐PF), etc. (Champtiaux et al. 2019; Wang et al. 2024). However, despite a large body of epidemiological evidence that smoking is not conducive to PF, the mechanism of smoking‐induced PF is still unclear. In this study, we found that significant inflammation and fibrosis occurred in the lungs of tobacco‐exposed mice. Mechanistically, smoking upregulated the expression levels of the methyltransferase MECP2 proteins, thereby transcriptionally inhibiting POT1 expression through a DNA methylation manner and a MECP2‐FOXP2 interaction manner. Low POT1 expression also led to the senescence of AT2 cells and the secretion of large amounts of SASP, thereby activating fibroblasts. Restoration of POT1 significantly alleviated smoking‐induced PF.

Our study showed that cell senescence was present not only in smoking‐induced PF mice but also in Bleo‐induced PF mice, suggesting that cell senescence may be a common feature of different types of PF. This finding is consistent with previous studies (Zhou et al. 2019). Further investigation showed that cell senescence was mainly present in AT2 cells in PF tissue. We also found obvious cell senescence in endothelial cells. However, unlike AT2, endothelial cell senescence did not increase further in Bleo‐induced PF mice, whereas AT2 cell senescence continued to rise. Therefore, we believe that AT2 cell senescence is the primary cell involved in smoke‐induced PF. This finding will also help elucidate the pathogenesis, diagnosis, and treatment of other smoking‐related respiratory diseases involving AT2 cell damage, such as lung cancer and COPD. Our findings also have realistic clinical implications, suggesting that we should try not to smoke, including staying away from passive smoking, whether we are healthy people or patients with PF.

Telomeres have the critical role of protecting chromosomes from degradation by nuclease, preventing chromosomes from fusing, and ensuring complete replication of chromosomes (Blackburn 1991). The length of telomeres determines the number of cell divisions and controls the process of cellular senescence and death, affecting the length of human life (Whittemore et al. 2019). We next found that POT1 expression was significantly reduced in smoke‐exposed AT2 cells. Induction of POT1 expression in AT2 cells then effectively attenuated smoke‐induced cellular senescence. These results suggest that reduced POT1 may be an important target for AT2 cell senescence. In addition, we found that low POT1 expression was regulated by methylation and that smoking increased the expression of several methyltransferases in AT2 cells. Therefore, methylation regulation should be fully considered in other diseases caused by low POT1 expression. We also examined DNA methylation levels in the CpG island region of the promoter region of the POT1 gene after smoking induction. It was found that smoking leads to significant changes in DNA methylation in the *POT1* CpG island region. Consistently, the use of a 5‐Az demethylation reagent significantly restored POT1 expression. Notably, we found that the CpG island of POT1 does have transcriptional activity through dual‐luciferase reporter experiments, further supporting the idea that smoking regulates the transcriptional level of POT1 by modulating the methylation of its CpG island region. To find the transcription factor that can bind to the CpG island, we then identified FOXP2 as a positively regulated transcription factor of POT1 by JASPR software prediction and confirmed it by ChIP experiments. Interestingly, among the many smoking‐activated methyltransferases, we found that the FOXP2 protein was able to bind specifically to the MECP2 protein, thereby inhibiting the binding of FOXP2 to the POT1 promoter, ultimately leading to a reduction in the level of transcription. Here, we report for the first time a smoking‐stimulated pattern of low POT1 expression. Joseph Kelich et al. reported that POT1 (L259S) is a pathogenic driver of familial IPF by inducing telomere loss, lagging strand defects, telomere‐induced DNA damage, and premature senescence with G1 arrest. We then examined the effect of CSE on the POT1 (L259S) mutation in primary AT2 cells and found that no POT1 (L259S) mutation occurred in primary AT2 cells during up to 2 months of continuous CSE stimulation. Therefore, we concluded that smoking may not exert its pro‐senescent effects by inducing mutations in the POT1 gene. However, it is more likely to result from induced transcriptional overexpression of POT1 (Figure S7). Senescence is regulated by multiple signaling pathways (Li et al. 2023; Zhang et al. 2024). To elucidate the senescence signals regulated by POT1, we examined the expression levels of p‐ATM, p‐ATR, and P53 and found that all of these pathways were significantly upregulated after POT1 knockdown. We also treated POT1 knockdown AT2 cells with p‐ATM and p‐ATR inhibitors and found that both inhibitors were effective in reversing senescence. This finding highlights the anti‐aging role of POT1 and provides a direction for clinical intervention in PF patients with deficient POT1 activity.

Fibroblasts are the primary effector cells in PF. Upon sensing tissue damage, fibroblasts proliferate, differentiate, and migrate to repair the tissue. However, persistent injury leads to excessive repair and structural rearrangement of lung tissue, culminating in fibrosis and respiratory failure (Shi et al. 2022). We found that senescent AT2 cells that knock down POT1 secrete large amounts of proinflammatory and profibrotic SASP components. When fibroblasts were treated with AT2 cell culture medium derived from si‐POT1‐pretreated AT2 cells, fibroblast proliferation, differentiation, and migration were significantly enhanced, and extracellular matrix such as collagen and other components were abundantly synthesized.

In conclusion, we report the role and mechanism of smoking in promoting PF. Briefly, as shown in the graphical summary, our study demonstrated that cigarette smoking induced the downregulation of POT1 expression by promoting DNMT expression. Low expression of POT1 may activate phosphorylation and activation of ATM, ATR, and p53 through DNA damage, and these pathways further cause senescence of AT2 cells. Senescent AT2 cells also secrete proinflammatory and profibrotic SASP components that promote fibroblast proliferation, activation, migration, and collagen deposition. Regulation of cellular senescence by POT1 is expected to provide a new primary therapeutic target for PF.

## Author Contributions

M.S., P.W., and W.W. conducted experiments and collected and mined data. Z.S. analyzed and interpreted data. J.S., H.C., X.C., J.C., and J.W. analyzed the results. X.S. designed the conception and experiment, supervised the study, and wrote the manuscript. All authors read and approved the final manuscript.

## Ethics Statement

All animals' experiments were operated according to the guidelines approved by the Institutional Animal Care and Use Committee of Fudan University (Approval no. FE20002). All experiments involving human patients were conducted according to the ethical policies and procedures approved by the ethics committee of the School of Life Sciences of Fudan University (Approval no. KY2023‐015).

## Consent

All participants signed informed consent forms and were notified about the study before participating.

## Conflicts of Interest

The authors declare no conflicts of interest.

## Supporting information


Data S1.

## Data Availability

The data of scRNA‐seq and snRNA‐seq were deposited under the NODE Accession ID: OEZ00021306.
