# Supplementary material for: Smoking Promotes AT2 Cell Senescence and Exacerbates Pulmonary Fibrosis by Downregulating POT1 via Integratively Inducing CpG Methylation and MECP2‐Mediated FOXP2 Transcriptional Binding Inhibition
Source: Aging Cell. 2025 Jul 20;24(10):e70174. doi: 10.1111/acel.70174 (PMC12507393; doi:10.1111/acel.70174)
Supplement: Supplementary file 1 — Data S1. [file ACEL-24-e70174-s001.pdf]

Smoking promotes AT2 cell senescence and exacerbates pulmonary fibrosis by downregulating POT1 via integratively inducing CpG methylation and MECP2-mediated FOXP2 transcriptional binding inhibition

Supplemental Figures and Tables

Supplemental Figures

Supplemental Figure S1

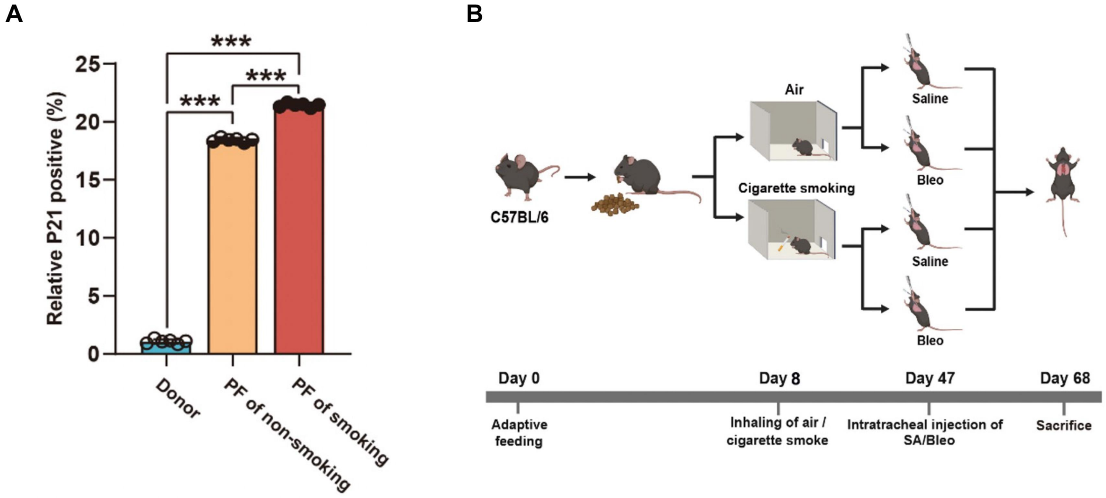

Supplemental Figure S1. The protein levels of P21 in lungs and the schematic diagram of smoking- and bleomycin-induced mouse PF. (**A**) The protein levels of pulmonary P21 in healthy donors, PF of non-smoking, and PF of smoking patients by IHC analysis. N = 6 per group. (**B**) The schematic diagram of smoking- and bleomycin-induced mouse PF. Data represented as median±SEM. Data in A was evaluated by one-way ANOVA (Tukey test). ***P < 0.001.

Supplemental Figure S2

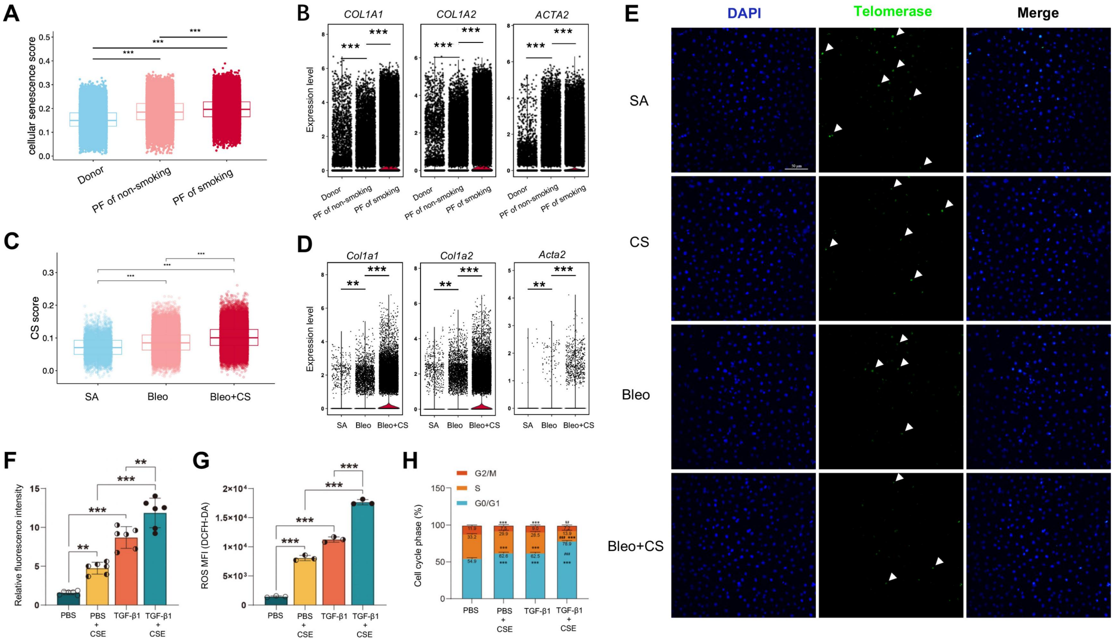

Supplemental Figure S2. Cigarettes induced cell senescence *in vivo and in vitro*. **(A−B)** The cellular senescence scores and expression of fibrosis-related marker genes in each group by scRNA-seq analyses in human PF patients. **(C−D)** The cellular senescence scores and expression of fibrosis-related marker genes in each group by snRNA-seq analyses in PF mice. **(E)** qFISH images show the telomere lengths of AT2 cells from mouse PF models. **(F−G)** The relative fluorescence activity by ROS staining and flow cytometer analysis in AT2 cells after TGF-β1 and CSE treatment. **(H)** Quantification of the cell cycle phase after AT2 cells were treated with TGF-β1 and CSE. Data represented as median±SEM. Data was evaluated by one-way or two-way ANOVA (Tukey test). $^{**}P < 0.01$, $^{***}P < 0.001$.

Supplemental Figure S3

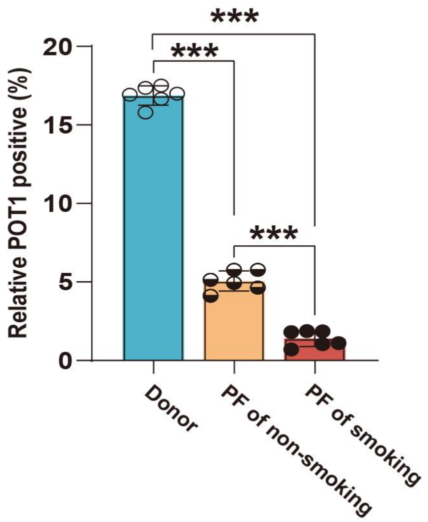

Supplemental Figure S3. The protein levels of pulmonary POT1 in healthy donors, PF of non-smoking, and PF of smoking patients by IHC analysis. N = 6 per group. Data represented as median±SEM. Data was evaluated by one-way ANOVA (Tukey test). ***P < 0.001.

Supplemental Figure S4

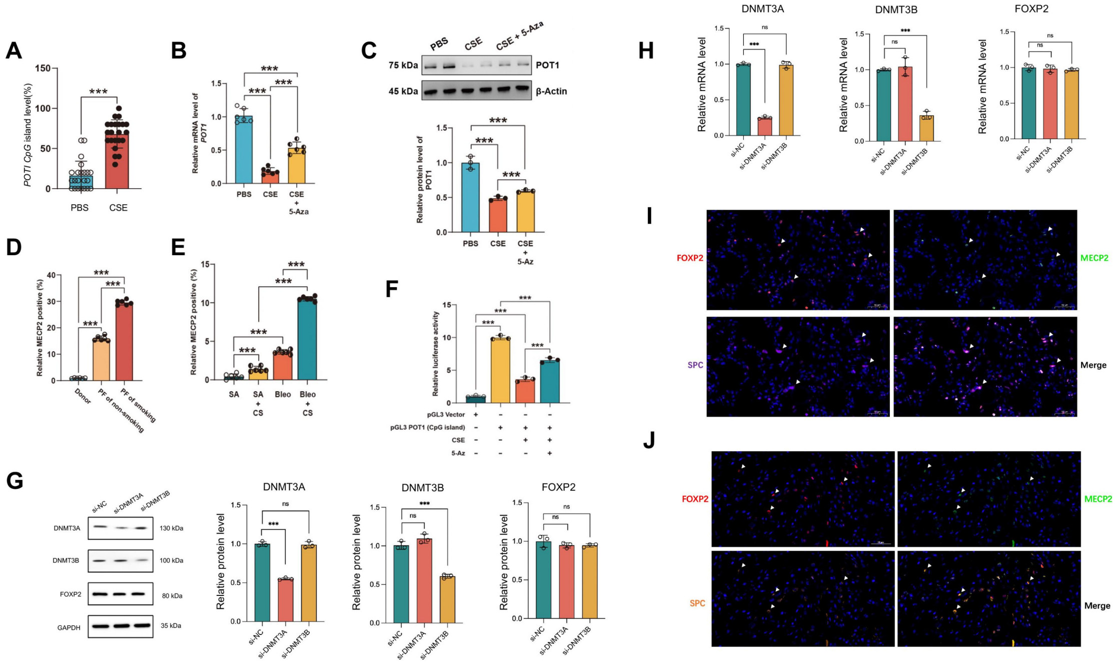

Supplemental Figure S4. CSE transcriptional inhibits *POT1* by inducing CpG DNA methylation of *POT1*. (**A**) The methylation percentages of *POT1* CpG island with CSE treatment. (**B**) The mRNA levels of POT1 after CSE and 5-Aza treatment. (**C**) The protein levels of POT1 after CSE and 5-Aza treatment. (**D**) The protein levels of pulmonary MECP2 in healthy donors, PF of non-smoking, and PF of smoking patients by IHC analysis. N = 6 per group. (**E**) The protein levels of pulmonary MECP2 in mouse PF models by IHC analysis. N = 6 per group. (**F**) The relative luciferase activity of *POT1* CpG island. **(G−H)** The expression of FOXP2 after knockdown of *DNMT3A* and *DNMT3B*. (**I−J**) Immunofluorescence staining shows the co-localization of MECP2 and FOXP2 in human (H) and mouse (I) lung tissue. Data represented as median±SEM. Data in A was evaluated using the Student's t-test. Data in B−F were evaluated by two-way ANOVA (Tukey test). Data in G was evaluated by one-way ANOVA (Dunnett-t test). *P < 0.05, **P < 0.01, ***P < 0.001.

Supplemental Figure S5

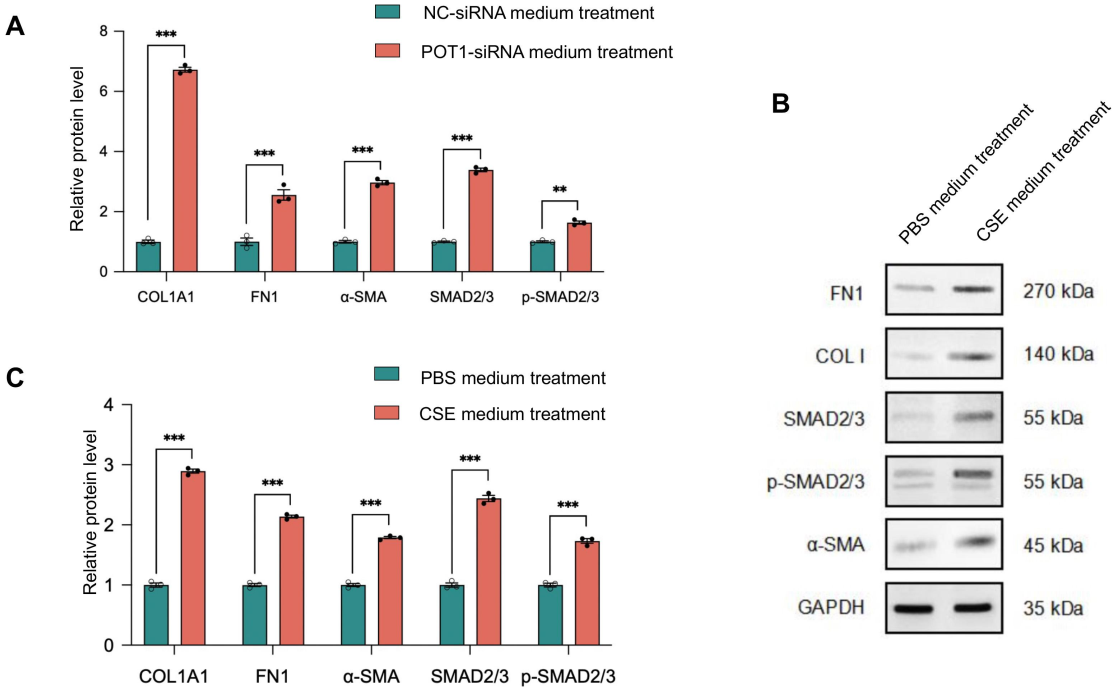

Supplemental Figure S5. Senescent AT2 cells have pro-fibrotic function for fibroblasts. **(A)** The protein levels of fibrotic markers and p-SMAD2/3 in fibroblast cells treated with conditional medium from *si-POT1*-pretreated AT2 cells. **(B−C)** The protein levels of fibrotic markers and p-SMAD2/3 in fibroblast cells treated with conditional medium from CSE-exposed AT2 cells. Experiments were repeated three dependent times. Data represented as median±SEM. Data was evaluated by Student's t-test. *P < 0.05, **P < 0.01, ***P < 0.001.

Supplemental Figure S6

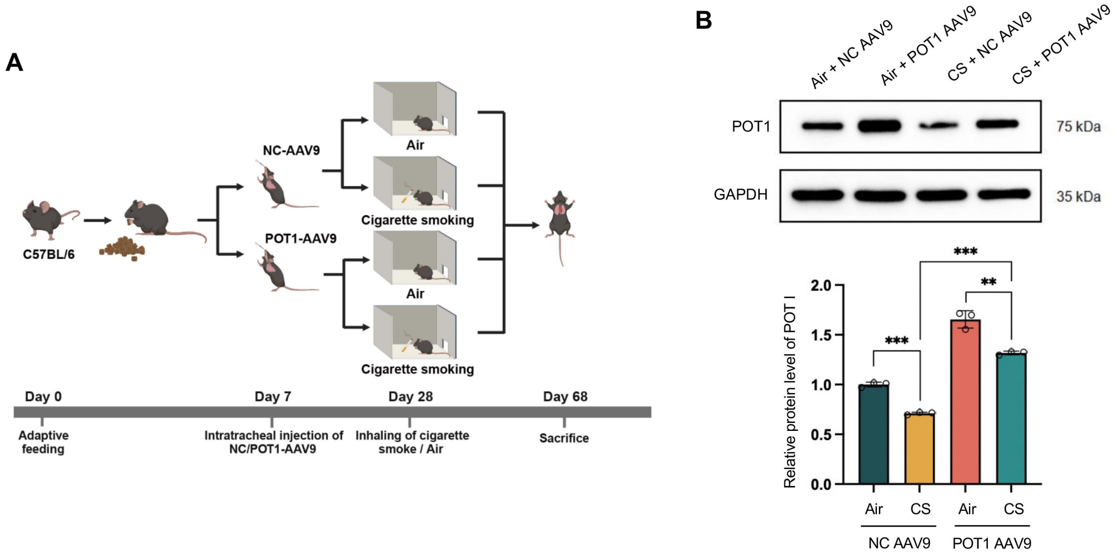

Supplemental Figure S6 The schematic diagram of smoking-induced mouse PF with AAV9-POT1 treatment and the expression of POT1 in sorted primary AT2 cells. (A) The schematic diagram of smoking-induced mouse PF with AAV9-POT1 treatment. **(B)** The expression of POT1 in sorted primary AT2 cells from each group of mice. Data represented as median±SEM. Data was evaluated by two-way ANOVA (Tukey test). *P < 0.05, **P < 0.01, ***P < 0.001.

Supplemental Figure S7

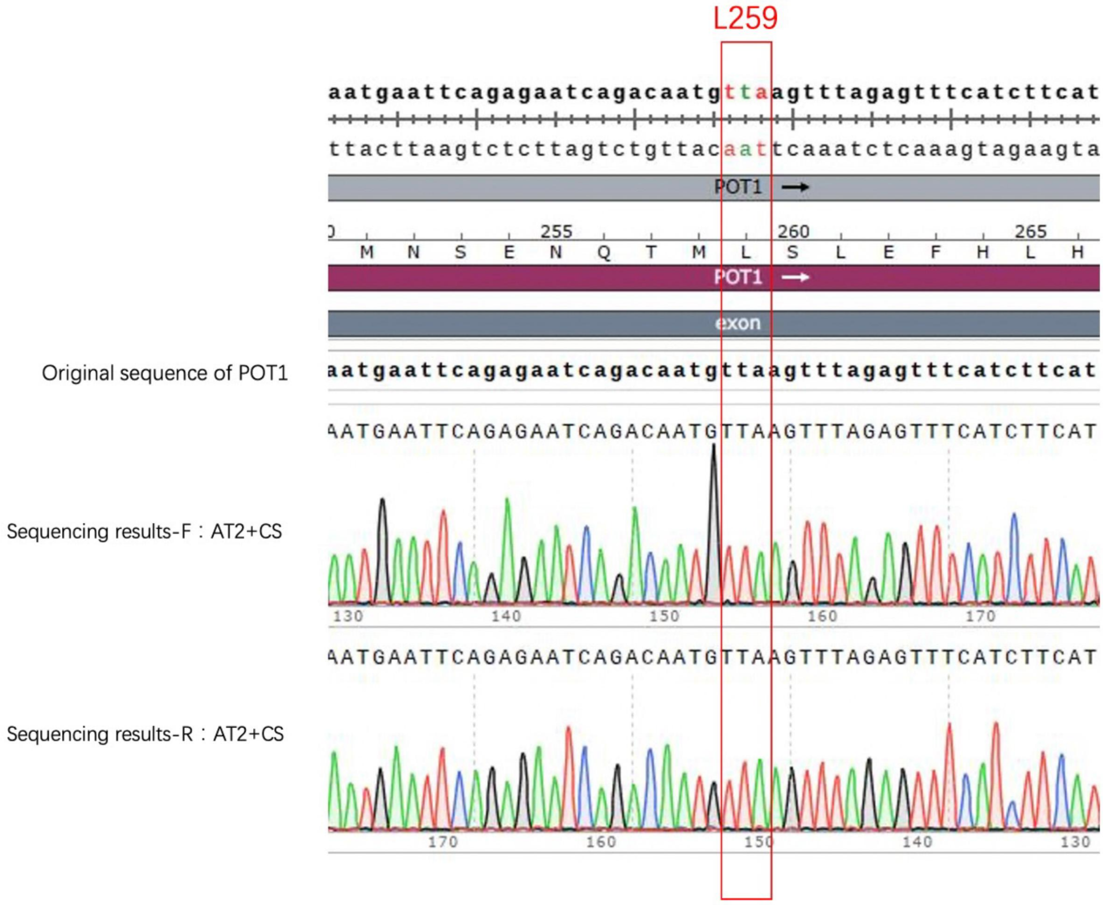

Supplemental Figure S7. DNA sequence detection of POT1(L259S) mutation in primary AT2 cells after two-month continuous CSE treatment.

Supplemental Tables

Supplemental Table S1

Clinical characteristics	non-Smoking IPF	Smoking IPF	Healthy individuals
(mean ± SEM)	(N = 8)	(N = 10)	(N = 6)
Age (years)	57.5±4.2	55.2±7.2	45.0±3.5
Gender			
Female	3	3	3
Male	5	7	3
Disease duration (years)	1.5 ± 1.5	1.9 ± 1.1	/
Medication history			
Pirfenidone	5 (62.5%)	6 (60.0%)	/
Nintedanib	3 (37.5%)	4 (40.0%)	/
Pulmonary fibrosis	100%	100%	/
Diabetes mellitus	/	/	/
Hypertension	/	/	/
Atherosclerosis	/	/	/
Familial dyslipidemia	/	/	/

Supplemental Table S2

Primer Sequence

Name	Human Primers Sequence (5'-3')
PCNA	F: CCTGCTGGGATATTAGCTCCA;<br>R: CAGCGGTAGGTGTCGAAGC
CDKN1A	F: CGATGGAACTTCGACTTTGTCA;<br>R: GCACAAGGGTACAAGACAGTG
CDKN2A	F: GGGTTTTCGTGGTTCACATCC;<br>R: CTAGACGCTGGCTCCTCAGTA
LMNB1	F: AGGAACCCGAGTTCAGCTAC;<br>R: CACGTCGAGGTCACCGAAAG
POT1	F: TTCCAGATTCCAGCATCAGAAGTTC;<br>R: TGAAGCATTCCAACCACGGATATG

DNMT1	F: AGAACGGTGCTCATGCTTACA;<br>R: CTCTACGGGCTTCACTTCTTG
DNMT3A	F: CCGATGCTGGGGACAAGAAT;<br>R: CCCGTCATCCACCAAGACAC
DNMT3B	F: AGGGAAGACTCGATCCTCGTC;<br>R: GTGTGTAGCTTAGCAGACTGG
MECP2	F: TGGGAAGCTCCTTGTCAAGAT;<br>R: TCGGATAGAAGACTCCTTCACG
TET2	F: ATACCCTGTATGAAGGGAAGCC;<br>R: CTTACCCCGAAGTTACGTCTTTC
COL1A1	F: GTGGGCAACGACTCTGGAC;<br>R: GCTTCGACATCAGCATTCCTCA
COL1A2	F: GATCACCCGAATGGCTATGAAT;<br>R: GGGGTCACAGTTGTCAATGTT
COL3A1	F: CACAACACGCTGTTCGGCTA;<br>R: CGATCCTGCATCTGTAAATCGC
FN1	F: AGAGGCATAAGGTTCGGGAAGAGG;<br>R: CGAGTCATCCGTAGGTTGGTTCAAG
ACTA2	F: AAAAGACAGCTACGTGGGTGA;<br>R: GCCATGTTCTATCGGGTACTTC
β-actin	F: CACAGAGCCTCGCCTTTGCC;<br>R: ACCCATGCCCACCATCACG
Name	Mouse Primers Sequence (5’-3’)
Col1a1	F: GCTCCTCTTAGGGGCCACT;<br>R: CCACGTCTCACCATTGGGG
Col1a2	F: GTAACTTCGTGCCTAGCAACA;<br>R: CCTTTGTCAGAATACTGAGCAGC
Acta2	F: CATCAGGGAGTAATGGTTGGAATGG;

	R: GTTCTATCGGATACTTCAGCGTCAG
Fn1	F: ATGTGGACCCCTCCTGATAGT;<br>R: GCCCAGTGATTTCAGCAAAGG
Cdkn2a	F: CAAGAGCGGGGACATCAAGACATC;<br>R: CACAAAGACCACCCAGCGGAAC
Cdkn1a	F: TCCTGGTGATGTCCGACCTGTTC;<br>R: ACGAAGTCAAAGTTCCACCGTTCTC
Lmnb1	F: AGACTTTGGGGGTTCATGTCA;<br>R: ATCGTCCCGTCTCCTTGTCA
Pcna	F: TTTGAGGCACGCCTGATCC;<br>R: GGAGACGTGAGACGAGTCCAT
Pot1	F: ATGGAGGTTGACCTGTTGGA;<br>R: TCCAGGTTGATGTTGAGGAC
β-actin	F: GGCTGTATTCCCCTCCATCG;<br>R: CCAGTTGGTAACAATGCCATGT

Supplemental Table S3

**Antibody**	**Cat#**	**Source**
FN1	A12977	Abclonal
COL I	A1352	Abclonal
ACTA2	GB111364	Servicebio
LMNB1	A4373	Abclonal
P21	A22460	Abclonal
GAPDH	GB15002	Servicebio
P16	A23882	Abclonal
PCNA	A0264	Abclonal
POT1	A1491	Abclonal
β-Actin	GB15001	Servicebio
TET2	A1526	Abclonal

DNMT1	A22455	Abclonal
DNMT3A	A2065	Abclonal
DNMT3B	A22658	Abclonal
MECP2	A24642	Abclonal
p-ATM	5883	CST
p-ATR	30632	CST
p-P53	9284	CST
H2AX	A2082	Abclonal
γH2AX	AP0687	Abclonal
SMAD2/3	5678	CST
p-SMAD2/3	8828	CST
Reactive Oxygen Species Assay Kit	S0033S	Beyotime
SA-β-Gal staining Kit	G1073-100T	Servicebio
